# Psychiatrists’ perceptions of conditions and consequences associated with the implementation of open notes: qualitative investigation

**DOI:** 10.1186/s12888-024-05845-6

**Published:** 2024-06-10

**Authors:** Julian Schwarz, Cosima Hoetger, Lena-Sophie Pluschke, Felix Muehlensiepen, Michael Schneider, Samuel Thoma, Tobias Esch

**Affiliations:** 1 Department of Psychiatry and Psychotherapy, Center for Mental Health, Immanuel Hospital Rüdersdorf, Brandenburg Medical School Theodor Fontane, Seebad 82/83, Rüdersdorf, DE Germany; 2 Faculty of Health Sciences Brandenburg, Brandenburg Medical School, Neuruppin, Germany; 3 Institute for Integrative Health Care and Health Promotion (IGVF), Faculty of Health/School of Medicine, Witten/Herdecke University, Witten, Germany; 4 Center for Health Services Research, Brandenburg Medical School, Rüdersdorf, Germany

**Keywords:** Open notes, Opennotes, Clinical notes, Electronic health records, Implementation, Online record access, Patient participation, Psychiatric care, Qualitative data

## Abstract

**Objective:**

In a growing list of countries, patients are granted access to their clinical notes (“open notes”) as part of their online record access. Especially in the field of mental health, open notes remain controversial with some clinicians perceiving open notes as a tool for improving therapeutic outcomes by increasing patient involvement, while others fear that patients might experience psychological distress and perceived stigmatization, particularly when reading clinicians’ notes. More research is needed to optimize the benefits and mitigate the risks.

**Methods:**

Using a qualitative research design, we conducted semi-structured interviews with psychiatrists practicing in Germany, to explore what conditions they believe need to be in place to ensure successful implementation of open notes in psychiatric practice as well as expected subsequent changes to their workload and treatment outcomes. Data were analyzed using thematic analysis.

**Results:**

We interviewed 18 psychiatrists; interviewees believed four key conditions needed to be in place prior to implementation of open notes including careful consideration of (1) diagnoses and symptom severity, (2) the availability of additional time for writing clinical notes and discussing them with patients, (3) available resources and system compatibility, and (4) legal and data protection aspects. As a result of introducing open notes, interviewees expected changes in documentation, treatment processes, and doctor-physician interaction. While open notes were expected to improve transparency and trust, participants anticipated negative unintended consequences including the risk of deteriorating therapeutic relationships due to note access-related misunderstandings and conflicts.

**Conclusion:**

Psychiatrists practiced in Germany where open notes have not yet been established as part of the healthcare data infrastructure. Interviewees were supportive of open notes but had some reservations. They found open notes to be generally beneficial but anticipated effects to vary depending on patient characteristics. Clear guidelines for managing access, time constraints, usability, and privacy are crucial. Open notes were perceived to increase transparency and patient involvement but were also believed to raise issues of stigmatization and conflicts.

**Supplementary Information:**

The online version contains supplementary material available at 10.1186/s12888-024-05845-6.

## Background

Electronic health records (EHRs) constitute a collection of electronic patient information; health care professionals involved in the patient’s treatment can view records via shared access across health care settings [1]. In several countries, patients can access these records using online record access (ORA). Importantly, the sharing process involves granting service users access to sensitive documentation including narrative visit reports or clinical notes, a practice commonly referred to as “open notes” [2–4]. Although the United States, Scandinavian countries, and several other European countries have successfully implemented open notes access to medical and psychiatric notes, Germany has not yet managed to do so even though the German Civil Code covers patients’ right to be granted access to their health information upon request since February 2013. In Germany, ORA was introduced in 2021, with each individual statutory health insurance provider having their own app-based platform featuring various degrees of technical functions. The “German Act to Accelerate the Digitalization of the Healthcare System” mandates that all health insurance companies offer ORA for laboratory results and an overview of prescribed medication from 2025 [5]. However, no timeline has been established for the implementation of open notes as part of ORA in Germany.

Open notes and particularly the sharing of clinical notes and narrative patient reports with vulnerable patient populations, including psychiatric patients, remains a controversial topic [6–8]. On the one hand, mental healthcare professionals (HCPs) believe open notes to result in a set of positive consequences beneficial to the therapeutic process via mechanisms of increased transparency, improved trust, and increased patient involvement [9]. HCPs believe that the communication and transparency resulting from open notes can have a positive impact on quality of care as well as patient empowerment [10]. Patients seem to agree with their HCPs; findings suggest that open notes are perceived as valuable treatment tools that could lead to improved patient-provider relationships which they considered to be critical to their treatment progress [11].

However, not all research findings highlight positive perceived consequences of implementing open notes. In fact, some HCPs believe the implementation of open notes to be of little use with less than 6% reporting that patients who read their notes exhibited better self-care and only 8% perceiving patients who read their notes to be more adherent to their medication regimen [12]. More concerning than a perceived lack of benefits of open notes are the perceived negative consequences anticipated by HCPs, some of which are linked to clinicians’ duty to protect patients’ wellbeing [13, 14]. Some HCPs are concerned that reading one’s clinical notes may result in unintended negative consequences such as patients experiencing confusion, anxiety, and a sense of stigmatization, all of which could negatively impact the therapeutic relationship and subsequent therapeutic progress [9, 15]. Research findings mirror such concerns with some patients reporting having experienced surprise, stress, and worry, and having felt judged as a result of reading their notes [16]. Particularly the sharing of notes with especially vulnerable patient groups including individuals suffering from severe disorders such as psychotic disorders or disorders that negatively impact patients’ ability to interact with others and establish and maintain trusting relationships such as personality disorders, presents an ethical dilemma [17–19].

In addition to patient-related consequences, clinicians believe the implementation of open notes to impact their professional workload [13, 14, 20–23]. Perceived consequences include a decline in the quality of clinical notes as HCPs may feel pressured to censor or alter their notes prior to sharing them with their patients or that they may prefer to not share some notes at all [6, 24]. Potential censoring was believed to occur due to a perceived risk of negative trade-offs which mental health professionals feared could result in reduced integrity and confidentiality for the all parties involved, which in turn could result in a loss of trust in the therapeutic relationship [22]. Another unintended negative consequence includes the risk of conflict as HCPs report concerns that gaining access to one’s clinical records may cause patients to disagree with the content of the notes and/or with their diagnosis [20].

HCPs also describe potentially problematic consequences of sharing notes containing information on traumatic experiences; these could result in issues associated with data protection and safety as the names and information of third parties cannot be protected [9]. However, it is possible that certain conditions could alleviate some of the potential negative outcomes associated with open notes if put in place prior to implementing open notes. For example, patients’ stress or anxiety resulting from reading their notes may be due to the language used in the notes. For example, 14% of participants in Blease et al. (2019) found their notes to be at least somewhat difficult to read, which supports findings from a systematic review suggesting that patients have a preference for simple language over medical terminology [9]. Additional, perceived stigmatization could be alleviated by using strategies including the use of respectful language, openness to discussing note content with patients, collaborative note taking, and highlighting of patients’ strengths and treatment progress [25]. The practice of collaborative note-taking constitutes a more advanced practice of open notes, also known as “OurNotes,” in which the patient and clinician both look at the computer screen and decide together what should be included in the note [26].

Findings regarding perceived consequences are mixed with some HCPs highlighting the benefits of increased transparency and patient involvement, while others express concerns that patients may experience psychological distress as a result of reading their notes [27]. Importantly, there is little research on what conditions HCPs believe need to be in place prior to implementing open notes to allow for a smooth transition from closed to open notes.

In order to better understand what factors can contribute to successful implementation of open notes in psychiatric care and what consequences HCPs anticipate as a result, we conducted interviews with psychiatrists. Our research questions included what conditions psychiatrists believed needed to be in place prior to implementing open notes, as well as anticipated changes that could occur as a result of the implementation of open notes, e.g. therapeutic dynamics, whether and to what degree patients’ diagnoses and severity of symptoms should influence access and use.

## Methods

### Study design

For the present study, a qualitative design was chosen. Semi-structured expert interviews were conducted using a sample of psychiatrists. Data collection was followed up using thematic analysis [28, 29]. For quality assurance purposes, the COnsolidated criteria for REporting Qualitative research (COREQ) checklist was used (see Appendix) [30]. Given that we exclusively interviewed experts and that our interview questions exclusively pertained to their views of an external topic, and neither patients nor patient data were used in the study, the institutional review board (IRB) at Witten/Herdecke University waived the need for ethics approval.

### Setting and sampling

The study participants were recruited in the federal states of Berlin, Brandenburg, and Baden-Wuerttemberg, Germany. We employed snowball sampling [24], a type of convenience sampling; the corresponding author (JS) approached psychiatrists directly and in person at his institution or via email. The individuals then shared the study information with colleagues in other clinics and settings. During this process, psychiatrists were approached directly by colleague JS and invited to provide the research team with the contact information of colleagues they believed would be interested in participating in our study. The research team then contacted these psychiatrists using the contact details provided by the psychiatrists (email or telephone) and invited them to participate in the study. In order to ensure that respondents had the sufficient work experience to understand the scope and consequences of open notes for the field of psychiatry, only psychiatrists with at least three years of professional experience were included. Informed consent was obtained via an electronic signature prior to conducting the interview. Study participants received financial compensation (50€).

### Data collection

The interviews were conducted from 10/2022 until 12/2022. We developed a semi-structured interview guideline informed by a previously published international literature review that outlined several research gaps [9]. In line with our central research question, our interview guideline contained the following key aspects: (1) previous experiences with record sharing, (2) conditions believed to be necessary for the successful implementation of open notes, (3) patient groups for whom open notes was believed to be particularly helpful or unhelpful, (4) physician-patient relationship, and (5) various forms of access and/or use in different treatment settings (outpatient, inpatient, etc.).The interview questions corresponding with these key aspects were designed in such a way that they inquired about both the conditions interviewees believed needed to be in place and the potential effects of the use of open notes on psychiatric care. The interview guideline is attached as supplementary material (see Suppl. Table 1).

A pretest was conducted with *N* = 2 psychiatrists. No issues were detected; thus, no changes to the interview guide were made and the pretest interviews were included in the analysis. Interviews took place via telephone or video call and were conducted by LP. Within the project, LP was responsible for data collection and assisted with the analysis. Additionally, no prior relationships existed between LP and the interviewees; thus, it is unlikely that the interviewees were influenced. As part of the interview, sociodemographic data were collected, and field notes were taken. Interviews were digitally recorded, transcribed, and de-identified. Participants were recruited until thematic saturation of the study results in themes and categories was achieved [29].

### Data analysis

Data analysis was conducted by the research team (LP, CH, JS) using thematic analysis to develop a system of categories [29]. The central two research questions of this paper were transformed into deductive main categories (necessary conditions and expected effects of open notes), which guided the data analysis. The subcategories were developed following both a deductive and inductive approach. Accordingly, central guideline topics can be found in the subcategories, such as “user groups” and “settings”, or “doctor-patient relationship”. Other subcategories, such as “time” or “resources and usability”, played a central role in the material and were therefore included as subcategories. The sub-subcategories were developed based entirely on the interview data, i.e. inductively. To increase the validity of the results and control for subjective bias, each transcript was coded by at least two researchers. In addition, the research team met regularly to resolve coding differences and agree on a category system. Communicative validation was established by presenting findings to the interviewees and inviting them to make comments and correct any information that they felt was a misrepresentation of their statements [31]. This validation process confirmed the results; thus, no changes were made. Analyses were performed using MAXQDA software (Verbi GmbH, Berlin, Germany).

## Results

A total of 20 psychiatrists practicing in the federal states Berlin, Brandenburg, Baden-Wuerttemberg, Germany, were invited to participate; 18 individuals who worked in the field of adult psychiatry agreed to participate. Sample characteristics are displayed in Table 1. The interviews lasted an average of 38 min (SD: 16.6 min).

**Table 1:** Sociodemographic data of the participants (*n* = 18)

Characteristics		Details
Age in years, m (SD); min.-max.		41.2 (9.3); 28–63
Gender, *n* (%)	female	8 (44.4)
	male	10 (55.6)
	diverse	0 (0)
Job position, *n* (%)	assistant physician	9 (50.0)
	senior physician	2 (11.1)
	chief physician	1 (5.6)
	consultant psychiatrist	6 (33.3)
Setting, *n* (%)^1^	inpatient	10 (55.55)
	day clinic patient	5 (27.78)
	outpatient	8 (44.44)
Professional experience in years, *M* (SD); min.-max.		11.8 (8.8); 3–37
Experience with paper-based note sharing, *n* (%)	yes	4 (22.22)
	no	14 (77.77)

1 Multiple answers were possible; M: mean value; SD: standard deviation

## Qualitative findings

Our findings reflect psychiatrists’ perceptions of what conditions need to be in place in order for the hypothetical implementation of note sharing to be a successful endeavor as well as psychiatrists’ views of anticipated changes resulting from said implementation.Table 2 outlines our findings by categories.

**Table 2:** Qualitative category system

Conditions perceived to be necessary for the implementation of open notes	Expected changes through open notes
**I. User groups and settings** • Useful across psychiatric diagnoses • Access restriction for particularly vulnerable groups experiencing severe symptoms • Useful across settings • Supporting the shift from inpatient to outpatient settings **II. Time** • Sufficient documentation time • Sufficient time to debrief patients after drafting visit summaries **III. Ressources and usability** • Simple user interface • Integrating open notes with current documentation processes **IV. Legal considerations and data protection** • Adapting data protection guidelines currently in place to meet requirements for patient use • Protection against access by unauthorized third parties	**I. Documentation** • Patient-centered content and language • Improved quality and increased scope of documentation • Protection through closed notes **II. Treatment processes** • Positive and negative impact of open notes on patient safety • Open notes can serve as a treatment guide, resulting in shorter treatment processes while still allowing for long-term benefits of treatment • Increased patient participation through increased health literacy • Conflicts can be prevented by discussing note content with the patient **III. Physician-patient interaction** • Increased self-reflection among physicians resulting in positive change of attitude • Balancing power hierarchies between psychiatrists and patients • Possible changes in the therapeutic relationship

### Conditions perceived to be necessary for the implementation of open notes

The conditions that psychiatrists in our sample believed should be met in order to ensure successful implementation of open notes can be divided into four categories (I-IV).

### User groups and settings

Psychiatrists stated that in order for the implementation of open notes to be successful, note sharing had to be beneficial and worthwhile for patients. Some interviewees believed open notes to be generally useful for patients regardless of their diagnoses.

Other interviewees perceived open notes as less useful or feasible for some patient groups, with some psychiatrists expressing concerns about whether certain patient characteristics, such as age, severity of illness, and digital health literacy could impact patients’ ability to access their notes online. However, interviewees did not name any definite characteristics of patients for whom open notes were perceived as less helpful; psychiatrists mostly leaned towards deciding on a case-by-case basis:

“No, there’s not any specific group [for which open notes is a particularly good fit]. I’d decide on a case-by-case basis. I can think of three or four patients with the same diagnosis that I have a different stance on [using open notes].” (P5).

Additionally, interviewees discussed granting access to open notes based on individual needs. Psychiatrists suggested that patients undergoing acute phases of their disorder should have their access temporarily revoked while suffering acute symptoms, but should regain access once stabilized.

Interviewees emphasized the importance of adhering to a set of guidelines outlining specific criteria that could be used by clinicians when determining whether and when to revoke access; standardization of decision criteria as well as guidelines on the duration of access restriction was believed to be a critical step to ensuring that all patients were treated fairly and equally.

*“If at all possible, at first, you could just stick to descriptive notes, while the patients are really acutely ill. As things progress, we can increase the level of access.” (P11)*.

*“I imagine [access restriction] would be difficult to implement. I can’t just say [to a patient] at a certain point, okay, at this point, I no longer think that you are a good fit for open notes.” (P6)*.

As we had previously observed among responses to questions regarding different patient groups, interviewees also thought that the degree to which open notes should be used should be dependent on the treatment setting; especially the use of open notes in acute inpatient settings was met with skepticism, as difficulties were believed to arise as a result of the severe levels of stress and overstimulation experienced by hospitalized patients. Participants perceived hospitalized patients as particularly vulnerable; specifically, participants believed that using electronic devices to access treatment information could place additional strain on patients; additionally, patients misinterpreting the note contents could jeopardize their mental health further. However, despite the concerns voiced by some interviewees, several individuals in our sample highlighted positive aspects of open notes use across all settings, including acute settings.

“I think it’s important to meet patients with as much openness as possible during intake, even in acute inpatient settings. But I could imagine that it simply leads to more insecurity and more harm if all information is accessible [to patients], because the persons are often in situations where they cannot handle it [e.g., what happened prior to hospitalization] due to the severity of their psychopathology.” (P11).

Additionally, psychiatrists perceived open notes access as a valuable tool to ensure continued patient support and close monitoring despite calls from the healthcare systems’ legislators to move away from inpatient treatment settings towards outpatient care, assuming that a newly implemented open notes platform would entail a messenger function.

Open notes was believed to aid in improving patients’ adherence to medication regimens as access to notes was thought to be used by patients to look up prescription-related information; thus, patients’ medication safety was believed to be improved by using open notes to provide information on the correct dosages and times when the medication should be taken. Interviewees believed that by doing so, risks of incorrect dosing could be reduced. Additionally, providing patients with the option to look up notes from past sessions was thought to serve as a source of support for the patients; for example, patients could use such notes to go over previously discussed coping skills if they experienced difficulties recalling such skills during an acute situation. Thus, interviewees anticipated that their advice would be used via open notes during times while the clinician would not be available to answer questions.

*“Patients are told that they don’t need to come to the clinic on that day and that they should go ahead and go about their day, but [we also tell them] that they should stay in contact with the clinic. On Mondays, you see them during rounds, and you get to hear how things had gone [for the patient] outside of the clinic. During the shift from inpatient to outpatient treatment, [open notes are] a helpful add-on that can be very easily implemented.” (P2)*.

### Time

Time was believed to be a critical factor when considering the feasibility of open notes throughout treatment. Psychiatrists reported that they experienced difficulties documenting patients’ visits during the designated time slots. Interviewees believed that in order to use open notes sensibly, additional time would be required in order to write session notes suitable for patients.

Disadvantages would include [psychiatrists] having to invest more time for the documentation, and you need the time because you have to carefully consider your language and because you include more [information] into the documentation, especially when discussing recovery-oriented aspects, because your documentation is not only done for [the Medical Service of the Statutory Health Insurance Funds], but also for the patients.” (P10).

Interviewees highlighted the need to discuss the shared document with the patient as an additional time-related issue. Debriefing the patient was believed to be necessary in order to avoid misunderstandings and subsequent conflict. While discussing the documentation content was thought to increase patient safety; conversations surrounding note content were also perceived to require a considerable amount of additional time:

“But I actually think, as far as the practical implementation [of open notes] is concerned, it would require more time. More time for the documentation, where you simply put a little more effort into it, and also more time to discuss the documentation with the patients and to have the time that you need to do so. I see that as a very crucial problem.” (P10).

### Ressources and usability

Interviewees believed that in order to ensure open notes’ usability, several patient portal functions would need to be in place prior to using open notes. Psychiatrists discussed several solutions to the anticipated difficulties:

“I think that most importantly [the patient portal] should be clearly structured and easy to use, with functions being put in place for medication plans, progress documentation and forms. And prescriptions would be important, too. All patients should be able to use it, and authentication should be secure, yet easy to use.” (P7).

“Maybe [the patient portal] can also be a way through which the patient can ask a question [about their notes] beforehand or something and then I as the psychiatrist should receive notification of that.” (P13).

Compatibility of a new open notes program with the program already established at the clinic was perceived to be critical. Participants expressed concern about having to operate two programs at once should open notes be implemented. In order to facilitate a smooth transition to implementation and use of open notes, psychiatrists thought it best to install open notes as an additional function within the current documentation system, thus not making it necessary for practitioners to adapt to a new program or use two programs at the same time.

*“It would be great if [open notes] could be integrated into our hospital’s internal information system. So that you wouldn’t have [to use] an additional program and you wouldn’t have to move [any information] back and forth.” (P15)*.

### Legal considerations and data protection

Participants thought that in order to establish open notes in clinical practice, general legal issues and data protection details would have to be considered beforehand. On the one hand, interviewees expressed concerns about whether secure storage of patient-related data could still be ensured when data would be accessed through an online portal by third parties located outside of the clinic (e.g. patients accessing the portal from home). On the other hand, participants believed that the option of reading one’s clinical notes could benefit psychiatrists from a legal standpoint; patients accessing their notes would be able to detect errors and/or discrepancies and notify their psychiatrist who could then correct the issue. The quick turnaround in error detecting and problem solving was believed to reduce treatment errors and prevent subsequent legal issues.

*“I think [open notes] can somewhat protect [psychiatrists] in the legal sense, so when patients are aware that I’m writing [my notes] in here and they can read them, [the patients] can just go ahead and [let me know] right away if there is an issue, whatever [the issue] may be.’ Those kinds of legal issues mostly occur when you’re dealing with [paper] charts.” (P15)*.

Data protection details must also be considered when dealing with patients disclosing information to third parties. Some patients were believed to be at risk of disclosing information as a result of their condition which would in turn prevent them from grasping the potential unintended impact of their actions. In the likely case that the individual with whom the information is shared has not undergone data protection training, there is a risk that sensitive information could be shared in ways that may be harmful to the patient. Participants emphasized that this risk necessitated that utmost attention be paid to data security particularly when working with psychiatric patients rather than somatic patients.

“No, I’m already hesitant when it comes to patients being able to access [their clinical notes]. We have patients who post their credit card and bank information on Twitter because of the severity of their condition; in this specific case [open notes], they could potentially read the content of their notes to their friends or share it on Facebook.” (P2).

“It’s possible that someone just logged into [the patient portal] via their cell phone, left [the device unlocked], and then someone else walks up and reads [the information on the patient portal]. It’s difficult for me to imagine how to sufficiently prepare [open notes for psychiatric patients].” (P3).

## Expected changes through open notes

Anticipated changes believed to take place as a result of the implementation of open notes are presented in three categories (I-III) with 13 sub-themes.

### Documentation

The interviewees believed that the records of documentation created for the treatment team and the billing department of German statutory health insurance companies were predominantly focused on patients’ deficits. One participant believed that open documentation held the potential of creating inner conflicts among practitioners; the interviewee believed that psychiatrists were being nudged to emphasize patient deficits to a degree that may not reflect reality while simultaneously feeling obligated to accurately document the current condition of the patient.

“We are being indirectly encouraged by the health insurance companies and practically directly encouraged by senior physicians and chief physicians to make people look much worse [i.e., sicker] in the documentation than they actually are in order to avoid case reviews by the medical service of the health insurance companies afterwards or even having [financial reimbursement for a patient’s entire treatment] withdrawn during case review, [which results in providers not receiving any reimbursement for the treatment]. How are we supposed to communicate to our patients: “I’m going to write down that you’re doing terribly, but obviously I know that you’ve made fantastic progress. But if I actually write that everything is going great, or that you have improved, the health insurance company is going to cash in on that.” (P9).

Additionally, the introduction of open notes was considered to be an opportunity to bring to light information previously omitted or minimized in the documentation, such as pieces of information that interviewees believed could potentially cause conflict between patients and practitioners, or information that practitioners wished not to address as it contained personal criticism and/or criticism of their own work. Interviewees stated that with open notes, practitioners might feel compelled to document events as realistically as possible regardless of potential consequences.

*“Because you would take a different approach to documentation than before when it comes to things you didn’t write down; those would [now] come to light.” (P16)*.

Interviewees also anticipated that open notes would lead to changes in the language used in clinical documentation, and that open notes would make documentation sound more empathetic and appreciative of the patient than interviewees thought it to be the case already.

*“I am a fan of phrasing [my notes] in a way that allows the patient to feel appreciated and not belittled. The documentation should not be too critical or judgmental, but rather descriptive. And especially if the patient was reading along, I would pay all the more attention to that.” (P7)*.

Interviewees preferred having the option of parallel documentation for certain cases, some of which would then be made inaccessible to patients (“closed notes”). Some interviewees suggested that certain note content should either not be shared at all or should only be shared with patients only at a time later on, e.g. when dealing with cases of domestic violence or sexual abuse. Participants reported that sharing information that they had not previously discussed with their patients was particularly problematic, including diagnostic hypotheses or notes that are meant to be discussed with other team members first. Interviewees anticipated that such information could present a challenge as it might adversely affect the clinician-patient relationship when shared with the patient.

“For the most part, I finish my documentation prior to discussing it [with the patient]. When meeting the patient for the first time, I document [my observations], think about [my observations], and I bring the patient chart along to a team meeting where I consult my colleagues. Those are some difficulties that actually arise when you use progress reports as some kind of reminder for yourself, where you first scribble down some thoughts that you wouldn’t communicate [to the patient] right away. I would imagine that [the therapeutic relationship could suffer] especially when you’re dealing with critical questions regarding a patient’s behavior and you end up communicating those right away.” (P10).

### Treatment processes

Interviewees believed that open notes could allow for more treatment continuity, as issues discussed during one session could be carried over into the next session. Because of their perceived contribution to increased continuity, open notes were thought to hold potential to shorten overall treatment time as appointments could be used more efficiently.

*“I think [open notes] would help us pick up the thread during treatment sessions more quickly. Sometimes patients bring a list of topics to discuss, but that tends to be the exception. I can’t always keep track of what needs to be discussed in more detail during the next session, and I don’t always have time to look [at my notes prior to the next session]. If patients could look at the session notes [using open notes] before their appointment to see what was discussed [last time], we could [pick the conversation back up] and get to the important points more quickly.”*.

*“I could imagine that the entire treatment process wouldn’t be as long either. It’s possible that patients would benefit more from treatment which would lead to shorter treatment duration overall.” (P10)*.

Debriefing the patient on their open notes at the end of the session was believed to be an integral component of the treatment process. Interviewees believed that answering patients’ questions, addressing their insecurities, and clarifying any information that patients may be confused about was a critical step in preventing misunderstandings and subsequent conflict that could damage the therapeutic relationship. Interviewees expressed that the debriefing process should include clarification of information that had not been previously communicated to the patient, including unfamiliar medical terms.

*“Perhaps the question [should be asked], for example, whether [a patient] is abusing medications that can be addictive. I believe that something like that can lead to conflicts if you just put it in the notes without talking about it.” (P10)*.

Better understanding of one’s diagnosis was thought to promote patients’ health literacy which was in turn believed to be necessary to facilitate more active patient participation, i.e. shared decision-making:

*“Especially with [psychiatric] diagnoses, it’s not irrelevant at all [from the patient’s perspective] to know what you’ve been diagnosed with. And if you ask them, quite a lot of [patients] don’t know what [diagnosis] they have. It’s astonishing how many [patients] don’t know. I don’t think it’s appropriate to reduce a person to their disorder either, but I still think it’s useful [for patients] to know what diagnosis you’re being treated for. It’s somewhat beneficial to feeling more in control of your own treatment.” (P15)*.

However, interviewees were concerned that open notes could negatively impact patient safety. Reading the notes was thought to potentially result in retraumatization among patients or trigger distress when reading information about diagnoses that had not previously been discussed. Patients’ distress was in turn thought to lead to some patients dropping out of treatment.

“But [reading the notes] can also have negative consequences if there’s anything in those notes that could lead to, for example, defiance or the decision not to take the medication or dissatisfaction and frustration. I could imagine that this would also negatively impact the risk of treatment discontinuation.” (P14).

On the other hand, patients’ treatment quality and safety were thought to increase as a result of using open notes as a reminder or reference tool:

“Unfortunately, [without open notes], there is often unnecessary confusion that doesn’t get resolved until [the patient’s] next appointment. So I think it’d be great if patients could look up [the information] in their electronic record and make sure.” (P4).

### Physician-patient interaction

Psychiatrists believed that the therapeutic relationship would be influenced by the use of open notes in different ways. The sharing of notes was associated with a perceived increase of transparency, which in turn was thought to strengthen patients’ trust in their therapist. Also, participants found it likely that open notes could, at least to a certain extent, decrease the power imbalance between psychiatrists and patients. This shift was believed to occur as a result of patients reading notes that emphasize the patient and are comprehensible to them.

“So I actually think that [reading the notes] would be beneficial and lead to more transparency and trust, whereas all that secrecy around what’s actually in the notes often leads to skepticism and a lack of trust towards practitioners.” (P17).

“But I think [open notes] softens that hierarchy a little bit and because of the language that is used in the notes, you perceive the other person as an individual who can think independently, and who is autonomous and vulnerable.” (P10).

The interviewed psychiatrists perceived the fact that the contents of the open documentation could be perceived as offending for patients as a risk. These perceived offenses committed by one’s psychiatrist may cause the patient to perceive the relationship as damaged.

“I would always consider it a risk that people could get offended and that this could lead to a breakdown of the [therapeutic] relationship.” (P11).

## Discussion

### Summary of principal findings

The use of open notes in the field of German psychiatric practice raised multiple questions among our interviewees. Importantly, the interviewees had no previous experiences with open notes as they practice in Germany, a country in which open notes have not yet been established as a part of the German health data infrastructure, despite Germany’s civil code stating that patients are to be granted access to their health records upon request.

Psychiatrists expressed interest in the application of open notes as part of their practice and believed open notes to be an effective tool for patients regardless of their specific diagnosis. However, interviewees were also in favor of limiting or restricting access for patients currently undergoing acute phases of a psychiatric illness and/or during times of hospitalization resulting from disorder-related symptoms. Limiting patients’ access to notes was believed to minimize the risk of patients misinterpreting clinical notes and subsequent negative consequences, such as damage to the therapeutic relationship.

The feasibility of open notes was perceived to be related to time spent comprising notes. Psychiatrists believed that compiling notes in layman’s terms and answering patients’ content-related questions would likely require additional temporal resources; interviewees reported that initiating the use of open notes could lead to changes in documentation and treatment processes, patient-clinician interaction, and patient safety. While psychiatrists believed that in addition to increasing patient participation, open notes could present a chance to record information that may otherwise be overlooked or even forgotten despite being of importance to the patient. Concerns regarding data protection were discussed as well. Additionally, interviewees feared that patients may feel offended when reading the contents of their psychiatrists’ notes, which they believed could lead to conflict. Our findings highlight the need for careful consideration and planning prior to introducing open notes in psychiatric practice.

#### Potential restrictions of access

Participants discussed whether access to open notes should be withheld from patients who were diagnosed with certain conditions. Past research findings suggest that some psychiatrists feel opposed to the use of open notes among some patient groups, including individuals diagnosed with psychotic and personality disorders [24]. Our interviewees did not find the application of access restrictions for individuals with specific diagnoses helpful, but instead thought that the restriction of access during certain times would be helpful, e.g. during a time period when a patient exhibits increased vulnerability. Specifically, our interviewees suggested using some sort of temporary virtual access barrier that could be activated during particularly stressful time periods and deactivated when the patient improved. Moreover, interviewees raised the critical question as to how the term “acute” should be operationalized and how acute any given illness and its symptoms would have to be in order to justify restriction of access. Additionally, interviewees pondered what criteria should be used to establish a guideline that clinicians could adhere to when determining access and restriction thereof. Guidelines are already in place elsewhere; in order to prevent patients from being harmed by the content of their clinical notes, individuals receiving inpatient care in some Swedish regions are blocked from accessing open notes until 2 weeks after their discharge from the hospital [32]. Germany’s right to informational self-determination dictates that physicians are required to grant patients access to their notes (i.e., physicians do not “own” their notes; [33]). The option of imposing an embargo has been described elsewhere [27, 34].

However, there are reasons why granting or denying access based solely on one’s patient status (inpatient vs. outpatient) may be insufficient when seeking to prevent vulnerable patients from experiencing harm; for example, even patients receiving outpatient care may experience symptoms to a degree that would justify or even necessitate restricting open notes access as a means to minimize the risk of exacerbation of symptoms as a result of reading one’s notes. German civil code dictates that patients’ requests to review their notes may be rejected if there are “substantial therapeutic reasons” to do so; justification must be provided [35].

In turn, some individuals receiving inpatient care may experience an increase of personal responsibility and self-efficacy as a result of using open notes [9]. In the present study, participants did not at all address managing patients with more severe cognitive impairment. However, promising findings from several countries highlight the benefits of the use of open notes access by individuals assisting with the healthcare needs of elderly or cognitively impaired patients when the latter are unable to independently access and/or understand their open notes [36, 37].

#### Counteract misunderstandings

Interviewees expressed uncertainty about how to prevent or in some cases address misunderstandings caused by open notes and highlighted, in line with past research, several factors they thought contributed to complications. These factors included a lack of understanding of medical jargon, the discovery of previously undiscussed content in open notes, and practitioners making errors when documenting note content. The irritations and misunderstandings were in turn believed to have a detrimental impact on patient safety [21, 38]. As a potential solution, our interviewees proposed discussing the clinical notes at the end of each visit; interviewees believed that summarizing what had been discussed throughout the session allowed patients to correct any errors and/or confirm that what had been documented was indeed correct. An example of the aforementioned approach has already been implemented; OurNotes, a concept developed by the OpenNotes research initiative at Harvard University allows for patients and clinicians to record session documentation together, i.e. create an outcome protocol of the respective session [39, 40]. OurNotes was perceived to not only prevent misunderstandings but was assumed to have positive effects on patient engagement and serve as a means of adding structure to clinical sessions. On the other hand, interviewees expressed concerns that depending on the implementation, both OurNotes and providing the patient with a verbal summary at the end of each session would require additional time that our participants did not feel was available to them.

#### Data protection

Our findings suggest that psychiatrists feel uncertain whether their patients are able to use open notes responsibly and in a manner that ensures data protection. For example, psychiatrists were concerned that patients may forget to log out of their account, thus inadvertently granting third parties access to personal information. Those interviewed were also worried that patients experiencing severe symptoms may actually grant unauthorized individuals access to their account, leading to substantial negative consequences, the specific nature of which participants did not elaborate on further. However, risks of unauthorized third-party access were previously highlighted by Bärkas et al. [41], who found that unauthorized access to open notes was particularly prevalent among psychiatric patients. Our findings highlight the unanswered question on how to strike a balance between ensuring that patient data is sufficiently protected while simultaneously providing patients with a user-friendly platform, the latter of which likely determines whether and to what degree patients choose to use open notes.

#### Burden of documentation

Similar to past findings [12, 22, 36], our participants perceived the use of non-medicalized language as an additional burden. However, interviewees also acknowledged that medical documentation was an issue as documentation was not only used to communicate information to patients but also to other stakeholders [9]. Overall, four different functions of medical documentation were named in the results: (1) communication of information between practitioners, (2) informing the patient (open notes), (3) legal documentation and (4) proof of a service provided (for reimbursement purposes). Having to communicate the same information to different stakeholders with varied interests raised the question of how psychiatric documentation could be adapted in order to meet the needs of all parties involved. In order to ease the burden of clinicians, interviewees called for a shift in health insurance-related processes currently in place. In Germany, statutory health insurance companies utilize clinical notes to determine whether treatments rendered are justified and whether the treatment is completed within an appropriate time frame given patients’ clinical progress. If deemed necessary, health insurance companies may terminate reimbursement for services especially if patients are either determined to present with symptoms below the threshold necessary to cover their treatments or because a clinician’s documentation has failed to adequately convey a patient’s need for a specific treatment [42].

The task of meeting the needs of all stakeholders when writing notes could potentially be made easier by the introduction of artificial intelligence (AI). Improving physicians’ notes through the use of AI could work in terms of both patient-centeredness as well as time management (i.e. minimization of documentation burden). AI has received attention in the past; for example, studies show that AI-generated responses to medical questions could potentially be more empathetic and of higher quality than those written by medical professionals [43]. In fact, Blease et al. [44] propose that in the future, open notes could be co-documented by practitioners and chatbots; practitioners could continue using medical terminology which could be processed by the chatbot, resulting in information conveyed in layman’s terms that could then be verified by practitioners, and eventually be viewed by patients. Storing one official medical note and an additional, AI-modified version of the note that can be easily understood by patients would meet the needs of all stakeholders, thus addressing all four aforementioned purposes of documentation.

#### Closed notes and information blocking

Interviewees anticipated encountering difficulties when having to share previously personal notes related to patients’ diagnoses. A potential solution to this issue is the practice known as “closed notes”, i.e. the keeping of an additional set of documentation that is not shared with patients [45] or, alternatively, a delayed release of notes, i.e. imposing an “embargo” [27, 34]. The basis of closed notes is the perceived need of practitioners to write down certain preliminary notes or hypotheses for their own use or to share with colleagues before discussing them with patients. Perhaps because closed notes contradict the principle of openness and transparency, keeping a second set of unshared records remains a rather controversial topic in the mental health field. For example, in the U.S., psychotherapy notes are not to be shared and information blocking is allowed if doing so “…will substantially reduce the risk of harm” if there is danger to self or others or if there is a privacy exception (§ 171.201(a) p. 704) [46]. Nevertheless, a Delphi survey of international mental health experts concluded that the benefits of information blocking (such as protecting patients) is outweighed by a substantial potential for harm, including feelings of stigmatization [18]. Our interviewees noted that the use of closed notes should occur at the psychiatrist’s discretion.

### Strengths and limitations

To the best of our knowledge, our study is the first to investigate German psychiatrists’ attitudes regarding the use of open notes in clinical practice. However, the qualitative study could only reach a relatively small sample in the federal states of Berlin, Brandenburg, and Baden-Wuerttemberg; thus, our findings cannot and do not serve as a definite representation of psychiatrists practicing in other regions of the country. Additionally, although individuals working in professions other than psychiatry treat individuals living with mental health disorders and would have certainly added invaluable insight to our findings, another limitation is that our sample included only psychiatrists. We aimed to investigate the perceived importance of regulating open notes access for some of the most vulnerable patient groups who are often in the care of psychiatrists, e.g. as medication may be needed as part of their treatment. Importantly, given that open notes has not yet been established in German mental healthcare (apart from a single pilot project)[47, 48] our study only addressed attitudes surrounding the hypothetical implementation of open notes on psychiatric work. This limits the transferability of the results and should be supplemented by research into actual experiences of use once the innovation has been implemented in the healthcare system.

Other limitations include possible selection biases. Psychiatrists who agreed to participate in the interview may have been more open to the idea of open documentation and/or may have had a more positive attitude towards granting patients access to their notes. While only one of the interviewees stated outright that they rejected open notes, our interviewees’ critical questions about and extensive mention of potential barriers to open notes implementation in the field of psychiatry suggest that the research questions asked as part of our study were answered sufficiently thus reflecting the whole picture instead of positive or negatively skewed opinions.

A strength of our research is that criteria of reliability and reflexivity have been carefully considered and met in order to ensure that different researchers arrive at similar results and/or challenge and overcome underlying personal beliefs and biases. Thus, each individual interview was coded by multiple researchers (LP, CH, JS). In addition, the evolving category system was critically discussed and consented upon in regular meetings of the research team (LP, CH, JS). Finally, communicative validation was conducted by presenting the results to individual study participants, asking them to review them (JS). The feedback given was in turn taken into account when revising the results.

### Implications for future research

Our research was conducted in a country in which open notes are not yet part of the (psychiatric) health data infrastructure. In order to investigate actual real-life consequences for psychiatrists or their patients, open notes would first have to be established. However, psychiatrists’ expectations surrounding open notes have been thoroughly investigated. However, an important next step is to determine the accuracy of such expectations.

First, future research should investigate whether open notes and the subsequent changes of documentation do indeed place additional strain on psychiatrists’ temporal resources. Should these concerns be determined to be valid, research should investigate whether and to what extent patients’ benefit may justify additional efforts on psychiatrists’ parts. Beyond findings from previously published findings [49], health economic analyses could provide answers to these questions. For example, as a first step, potential differences in time spent on writing conventional vs. shared notes should be investigated by conducting comparison analyses. This question is particularly relevant for psychiatrists as past research has found that writing notes takes up the largest proportion of time spent on documentation [50].

Second, it should be examined whether and to what extent access restrictions for patients living with certain mental health conditions are necessary and/or helpful. Importantly, although found to be inconsistent, the attitudes of psychiatrists regarding notes restriction have been thoroughly investigated; our findings revealed that access restriction-related decisions should be made by both the psychiatrist and the respective patient. Future research should investigate what specific criteria (i.e. symptoms, diagnosis, current state) lead to a tipping point at which open notes no longer benefit a patient and pose a risk instead. Importantly, should consensus be reached that access restrictions should apply in some cases, criteria that are both feasible and unambiguous need to be established in order to allow for psychiatrists to implement and lift access restrictions in a way that benefits the patient. However, one important issue concerning access limitations remains to be addressed; the point of views of patients, patient advocates, and individuals directly providing support to patients (e.g. informal caregivers and/or loved ones supporting the patient) have received little attention so far.

Third, potential changes resulting from the implementation of open notes will likely necessitate some changes in the way that psychiatrists write and maintain their documentation in order to ensure that open notes are used effectively and profitably. However, this also includes consideration of how patients can be trained to maximize the potential of open notes, and how they can communicate their concerns should issues such as side effects arise. Findings derived from future research studies should inform the development of guidelines aiding the writing and reading of open notes for patients and practitioners.

Fourth, the aforementioned findings hint at the potential role of artificial intelligence (AI) in clinical settings. Perhaps all stakeholders involved could benefit from investigating whether and how AI can be utilized to create documentation that can be easily understood by patients [44]. If found effective, AI could potentially reduce psychiatrists’ workload while simultaneously reducing the risk of misunderstandings and perceived stigmatization among patients.

## Conclusions

Our findings revealed that psychiatrists’ perceptions of the implementation of open notes vary. While acknowledging the potential benefits of this socio-technical innovation for improving patient engagement, psychiatrists express concerns regarding possible negative effects of open notes on therapeutic relationships and patient safety. Additionally, it remains unclear whether patients with certain diagnoses and/or a certain severity of symptoms should be prevented from accessing open notes at times; however, most interviewees agreed that access should be restricted during acute phases of illness to minimize potential risks. This raises the questions of transparency in psychiatric care and whether transparency is an inherent right or rather a tool that can improve or, in cases where a patient is particularly vulnerable, hinder therapeutic progress.

The anticipated burden associated with providing additional and/or modified documentation highlighted by psychiatrists presents an issue that must be addressed when implementing open notes. Importantly, it should be considered that psychiatric documentation serves multiple purposes, including sharing information with other HCPs, informing patients, providing legal documentation, and providing proof of services provided. Agreeing on a method of documentation that meets the needs of all stakeholders involved will likely present a challenge. Of particular concern is the risk of misunderstandings resulting from notes being written in medical jargon and/or patients discovering diagnostic information in open notes that had not previously been disclosed to them. Possible solutions to this issue may include providing the patient with a verbal summary of each visit and/or the use of OurNotes. Lastly, data privacy remains challenging when granting patients access to open notes. Psychiatrists are uncertain whether patients can use open notes responsibly and whether there are privacy risks due to unauthorized third party-access.

### Electronic supplementary material

Below is the link to the electronic supplementary material.

Supplementary Material 1

Supplementary Material 2

## Data Availability

The qualitative data collected within the current study are not publicly available due to privacy restrictions but are available from the corresponding author upon reasonable request.

## Abbreviations

AI: Artificial intelligence
COREQ: Consolidated criteria for Reporting Qualitative research
EHR: Electronic health records
IRB: Institutional review board
ORA: Online Record Access
VA: Veterans Administration
