# Supplementary material for: Psychiatrists’ perceptions of conditions and consequences associated with the implementation of open notes: qualitative investigation
Source: BMC Psychiatry. 2024 Jun 10;24:430. doi: 10.1186/s12888-024-05845-6 (PMC11163720; doi:10.1186/s12888-024-05845-6)
Supplement: Supplementary file 1 — Supplementary Material 1 [file 12888_2024_5845_MOESM1_ESM.pdf]

Suppl. Table 1. Qualitative interview guideline

Category	***Questions***	***In-depth questions***
**Introduction**	The goal of today's interview is to learn more about what conditions psychiatrists like yourself believe must be in place so that open notes can be used successfully in psychiatric settings. We would also like to learn more about how you think using open notes could change the mental health field. You do not need to have any previous experience with open notes to answer these questions; we are interested in learning more about your beliefs and expectations as a psychiatrist. Electronic health records (EHR) offer the possibility of documentation and coordination of care within a single and/or multiple health care setting(s). Establishing patient portals allows for the streamlining of administrative processes, such as scheduling appointments or renewing prescriptions. Although EHRs contain various types of health-related information, a key feature associated with EHRs is patient access to clinical notes, narrative visit reports, and other clinician-entered patient data and findings. The practice of (mental) health record sharing between practitioners and patients is referred to as open notes.	
**Previous experience**	***Do you already have experience with patient access to files?***	Can you describe your experience with this?

Category	*Questions*	*In-depth questions*
**Conditions for implementation**	Imagine the following scenario: open notes are introduced in your practice/clinic, giving your patients the option to digitally access and read the information in their file, including any of the visit and progress notes you have written.<br><br>Can you share any thoughts or reactions you may experience when picturing this scenario?	
**User groups**	***Considering your patients' socio-demographic backgrounds and/or current diagnoses, what patient groups are <u>most</u> likely to benefit from accessing their patient records?***<br><br>***Considering your patients' socio-demographic backgrounds and/or current diagnoses, what patient groups are <u>least</u> likely to benefit from accessing their patient records?***	What factors influence the degree to which patients can benefit from reading their notes?<br><br>How can barriers to access be overcome?<br><br>Are there any risks you believe to be associated with

Category	Questions	In-depth questions
	When granting patients access to their patient files, should psychiatrists distinguish between patient groups based on their diagnoses and/or socio-demographic background? If so, what factors should psychiatrists consider prior to granting patients access? Which patient groups would be affected and how?<br><br>***Can you imagine that patients' understanding of their diagnosis/treatment/medication would change (improve/worsen) as a result of using open notes?***<br><br>***To what extent do you think treatment would change as a result of using open notes, and what types of changes would you anticipate? (e.g., language used in documentation, quantity/quality of documentation, time needed for documentation, responding to follow-up questions from patients).***<br><br>***From your perspective, to what extent might access to open notes affect the course of treatment?***	these patient groups accessing their records?<br><br>How can potential risks associated with record access be minimized?<br><br>Keywords: Information blocking, our notes, collaborative documentation<br><br>To what extent/why/why not?

Category	*Questions*	*In-depth questions*
		How to maximize benefits while minimizing risks
**Physician-patient interaction**	Do you think the relationship between you and your patients would change as a result of open notes and if so, how? (Trust/mistrust and openness/closure of patients influenced by shared documentation).	What advantages/disadvantages do you believe to be associated with this?
	To what extent might communication with patients change?	What advantages/disadvantages do you believe to be associated with this?
	To what extent could open notes change the role dynamics between you and your patients?	Co-decision making? What decisions are made by the patient/by the physician?

Category	*Questions*	*In-depth questions*
	To what degree do you think that some content could lead to conflict when read by patients? (e.g. information on diagnoses, medication, therapy goals)	What are the implications of a change in role dynamics?<br><br>How could potential conflicts be prevented?
**Treatment setting**	In what treatment setting (outpatient/day patient/inpatient; group vs. individual) do you think open notes access makes the most sense?<br><br>When granting patients access to open notes, should psychiatrists distinguish between different types of settings? If so, how could the degree of note access vary across settings?<br><br>How might access impact patient* safety? (Risk to patient well-being based on information read).	Example: In Sweden, open notes cannot be viewed by patients who are currently undergoing treatment in the intensive care unit as a result of judicial placement; only after release.
**Potential for improvement and wishes**	Considering what we discussed today, what is an ideal scenario of open notes use in psychiatric practice in regards to patient access? (regardless of the technical possibilities etc...)	

Category	*Questions*	*In-depth questions*
**Conclusion**	Were there any questions you would have liked me to ask that I didn't ask?<br><br>Is there anything else you would like to share with me?<br><br>Thank you very much for your time and have a nice day!	
