# Supplementary material for: Psychiatrists’ perceptions of conditions and consequences associated with the implementation of open notes: qualitative investigation
Source: BMC Psychiatry. 2024 Jun 10;24:430. doi: 10.1186/s12888-024-05845-6 (PMC11163720; doi:10.1186/s12888-024-05845-6)
Supplement: Supplementary file 2 — Supplementary Material 2 [file 12888_2024_5845_MOESM2_ESM.pdf]

Appendix. Consolidated criteria for reporting qualitative studies (COREQ): 32-item checklist

Developed from:

Tong A, Sainsbury P, Craig J. Consolidated criteria for reporting qualitative research (COREQ): a 32-item checklist for interviews and focus groups. International Journal for Quality in Health Care. 2007. Volume 19, Number 6: pp. 349 – 357

No. Item	Guide questions/description	Reported on Page #
Domain 1: Research team and reflexivity		
Personal Characteristics		
1. Inter viewer/facilitator	Which author/s conducted the interview or focus group?	Page 4
2. Credentials	What were the researcher's credentials? E.g. PhD, MD	N/A
3. Occupation	What was their occupation at the time of the study?	N/A
4. Gender	Was the researcher male or female?	N/A
5. Experience and training	What experience or training did the researcher have?	N/A
Relationship with participants		
6. Relationship established	Was a relationship established prior to study commencement?	Page 4
7. Participant knowledge of the interviewer	What did the participants know about the researcher? e.g. personal goals, reasons for doing the research	Page 4
8. Interviewer characteristics	What characteristics were reported about the interviewer/facilitator? e.g. Bias, assumptions, reasons and interests in the research topic	Page 17
Domain 2: study design		
Theoretical framework		
9. Methodological orientation and Theory	What methodological orientation was stated to underpin the study? e.g. grounded theory, discourse analysis, ethnography, phenomenology, content analysis	Page 3-4
Participant selection		
10. Sampling	How were participants selected? e.g. purposive, convenience, consecutive, snowball	Page 4
11. Method of approach	How were participants approached? e.g. face-to-face, telephone, mail, email	Page 4
12. Sample size	How many participants were in the study?	Page 4
13. Non-participation	How many people refused to participate or dropped out? Reasons?	Page 4
Setting		
14. Setting of data collection	Where was the data collected? e.g. home, clinic, workplace	Page 4

15. Presence of non-participants	Was anyone else present besides the participants and researchers?	N/A
16. Description of sample	What are the important characteristics of the sample? e.g. demographic data, date	Page 4<br>Table 1
Data collection		
17. Interview guide	Were questions, prompts, guides provided by the authors? Was it pilot tested?	Page 4
18. Repeat interviews	Were repeat interviews carried out? If yes, how many?	N/A
19. Audio/visual recording	Did the research use audio or visual recording to collect the data?	Page 6
20. Field notes	Were field notes made during and/or after the interview or focus group?	Page 6
21. Duration	What was the duration of the interviews or focus group?	Page 4
22. Data saturation	Was data saturation discussed?	Page 4
23. Transcripts returned	Were transcripts returned to participants for comment and/or correction?	N/A
Domain 3: analysis and findings		
Data analysis		
24. Number of data coders	How many data coders coded the data?	Page 4
25. Description of the coding tree	Did authors provide a description of the coding tree?	Page 8
26. Derivation of themes	Were themes identified in advance or derived from the data?	Page 4
27. Software	What software, if applicable, was used to manage the data?	Page 4
28. Participant checking	Did participants provide feedback on the findings?	Page 6
Reporting		
29. Quotations presented	Were participant quotations presented to illustrate the themes/findings? Was each quotation identified? e.g. participant number	Page 6-10
30. Data and findings consistent	Was there consistency between the data presented and the findings?	Page 6
31. Clarity of major themes	Were major themes clearly presented in the findings?	Page 11
32. Clarity of minor themes	Is there a description of diverse cases or discussion of minor themes?	Page 11-14
